# Charcot-Marie-Tooth Disease With Leukodystrophy: An Atypical Presentation

**DOI:** 10.7759/cureus.64335

**Published:** 2024-07-11

**Authors:** Ali Almarzooqi

**Affiliations:** 1 Neurology, Rashid Hospital, Dubai, ARE

**Keywords:** charcot-marie-tooth disease type x, white matter lesions, peripheral neuropathy, x-linked genetic diseases, new-onset seizure, cerebral leukodystrophy, charcot marie tooth disease

## Abstract

This case report presents a 23-year-old male diagnosed with Charcot-Marie-Tooth (CMT) disease, who exhibited additional neurological symptoms suggestive of leukodystrophy. The patient experienced recurrent episodes of slurred speech, imbalance, and a recent tonic-clonic seizure, prompting admission. Neurological examination and imaging revealed bilateral white matter changes, raising suspicion of leukoencephalopathy. Further investigations confirmed a nonsense mutation c.64C>T (p.Arg22*) in the gap junction beta 1 (GJB1) gene. This case underscores the complexity of Charcot-Marie-Tooth disease type 1 (CMTX1) with atypical central nervous system (CNS) manifestations, highlighting the importance of comprehensive diagnostic evaluations and a multidisciplinary approach to management.

## Introduction

Charcot-Marie-Tooth (CMT) disease is a well-known genetic disorder affecting the peripheral nervous system, primarily targeting motor and sensory nerves. In this case, we focus on X-linked Charcot-Marie-Tooth disease type 1 (CMTX1), the second most common form of CMT, accounting for 6.5% of all CMT patients [1]. CMTX1 is an X-linked dominant inherited disorder caused by mutations in the gap junction beta 1 (GJB1) gene on chromosome Xq13.1, which encodes connexin 32 (Cx32) [2]. Cx32 is associated with gap junctions and is expressed in Schwann cells and oligodendrocytes, typically presenting as a combination of demyelination and axonal neuropathy [3]. Electrophysiological assessment usually reveals reduced motor nerve conduction velocity, affirming its demyelinating nature. The majority of individuals experience onset within the first two decades of life [4]. Common indications include gradual distal muscle atrophy, weakness ("inverted champagne bottle"), reduced or absent reflexes, sensory dysfunction, and foot deformities such as pes cavus and hammer toes [4]. Transient central nervous system (CNS) dysfunction manifestations are now globally recognized [5]. In this case, we present a patient with associated leukodystrophy as seen on brain MRI.

## Case presentation

A 23-year-old male with a known case of CMTX1 for the last decade, diagnosed after his mother and following genetic testing, presented to our ED with his first ever sudden onset of slurred speech and tongue heaviness, generalized fatigue, and difficulty in ambulation due to limb weakness. His limb weakness quickly resolved by the time he was seen in the ED; however, he could not articulate words, and his history was taken from his stepmother. The patient was able to follow instructions during his examination. Most symptoms resolved 30 minutes after onset, leaving mild fatigue and a holocephalic headache after the patient underwent a computed tomography brain scan, which was reported as normal.

Upon further history, the patient denied dizziness before the episode, loss of consciousness, recent illness, fever, or medication use. He reported a similar speech impairment episode the day before that lasted about 30 minutes. He had a loss of consciousness episode a week ago with tonic posturing of all limbs followed by clonic activity, lasting about 5 minutes, during which he fell and had minor trauma to the occiput. His colleagues witnessed this episode; he denied pre-ictal symptoms for that event. Post-ictally, he felt fatigued and tender in the occiput and had never experienced such an episode before the episode resolved within a few minutes, as per the patient. He revealed a diagnosis of CMTX1, shared with his mother, and agreed to upload his genetic test results into our system.

The patient was awake, alert, and oriented to person, place, and time on examination. Speech was normal after 4 hours of reduced production and slurring. Cranial nerves were intact. The motor exam showed average muscle bulk without fasciculations, decreased muscle tone, and no abnormal involuntary movements. Strength was 5/5 per the Medical Research Council scale, except for reduced grip in the right hand and bilateral foot dorsiflexion at 2/5, with bilateral fine postural tremors in the hands. The sensory exam showed a mild reduction in pinprick and touch sensation bilaterally in the feet. Deep tendon reflexes were absent. Coordination tests were normal bilaterally. Gait was mildly affected due to weak dorsiflexion, manifesting as a subtle steppage gait.

The patient was investigated, and his blood work-up included a complete blood count, creatinine, urea and electrolytes, serum troponin T level, liver function enzymes test, erythrocyte sedimentation rate, C-reactive protein, serum homocysteine levels, creatine phosphokinase test, iron study, glycated hemoglobin (HbA1c), fasting lipid profile test, thyroid function test, vitamin B12 level, thrombophilia factors test, rheumatoid factor, cyclic citrullinated peptide antibody test, anti-cardiolipin blood level, anti-beta-2 glycoproteins level test, anti-nuclear antibody test, anti-neutrophil cytoplasmic antibodies test, anti-double stranded DNA test, phosphatidylserine antibodies test, lupus anticoagulant test, extractable nuclear antigen profile blood test, and lipoprotein (A) test. All the tests came back within an acceptable range.

**Figure 1:**
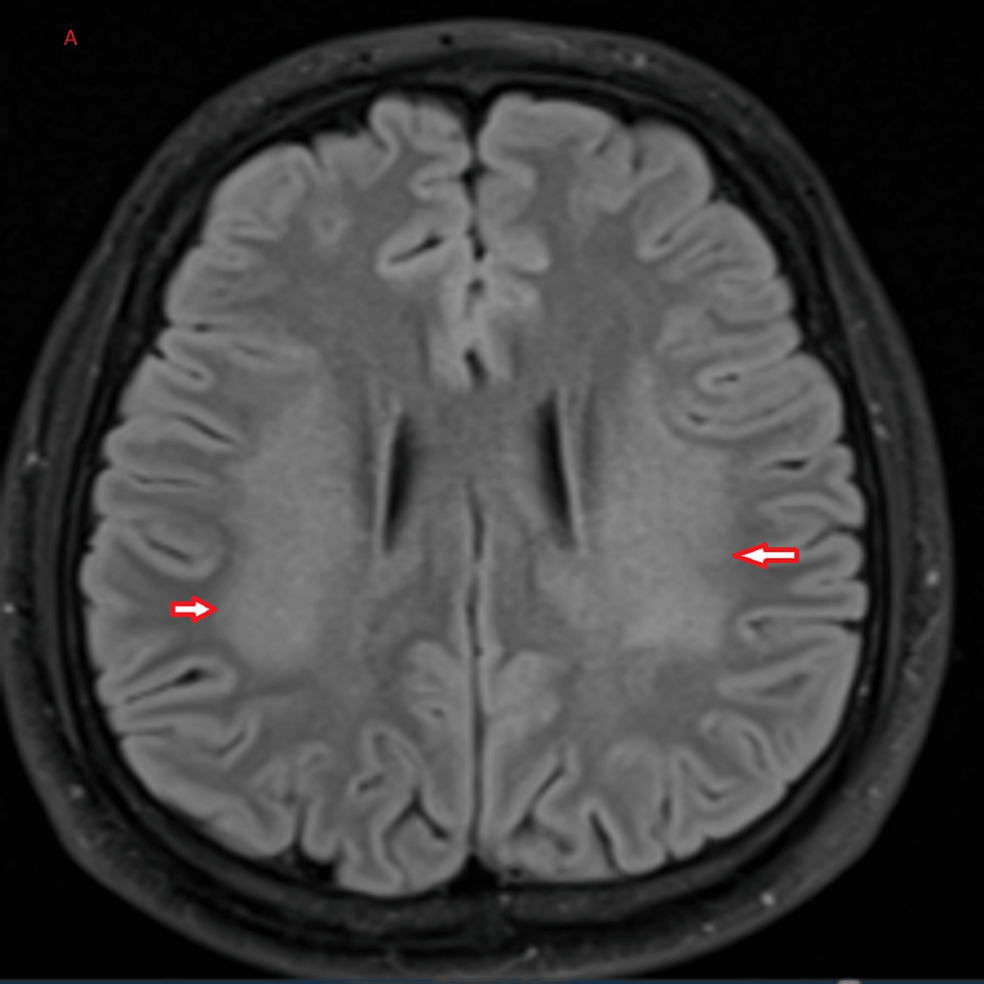
FLAIR hyperintense lesions. The FLAIR MRI sequence, axial cut, shows white matter hyperintense lesions, as indicated by the arrows. FLAIR: Fluid-attenuated inversion recovery.

**Figure 2:**
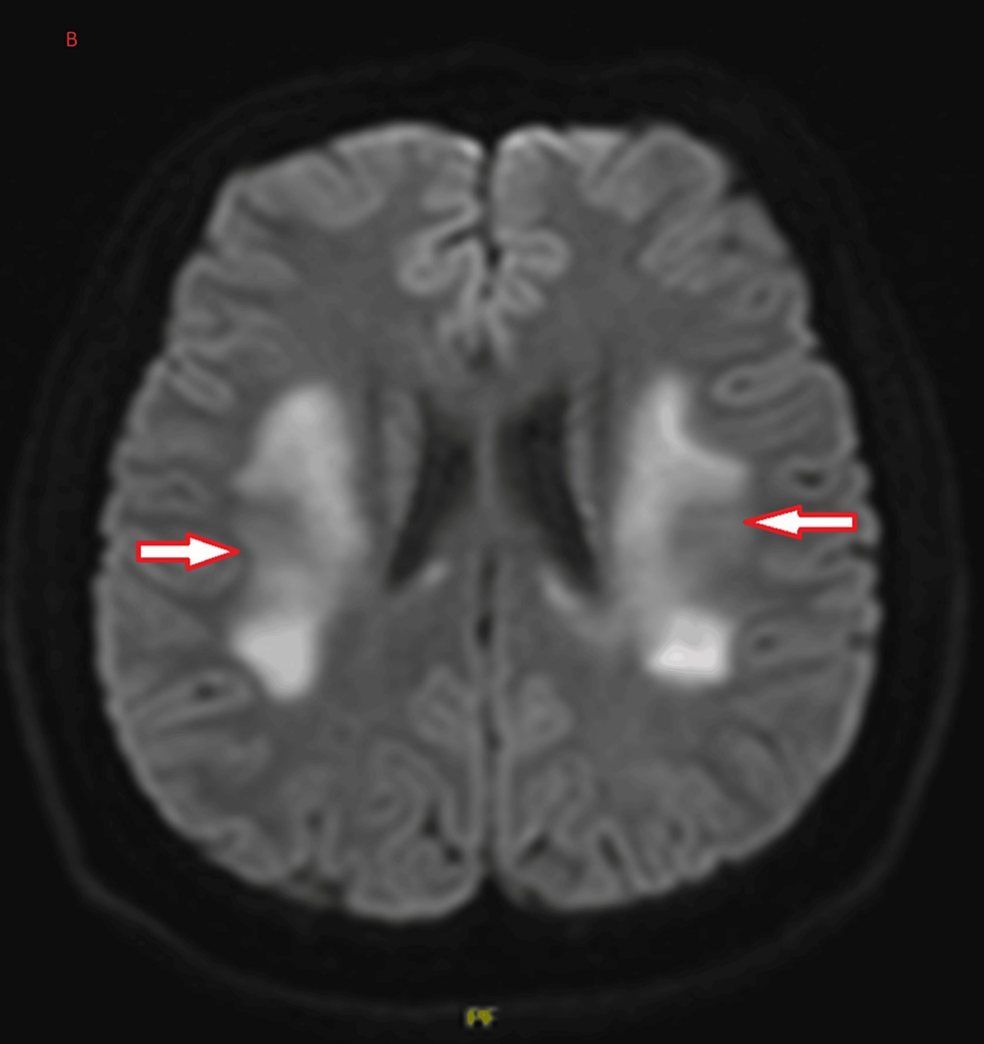
DWI hyperintense lesions. The diffusion-weighted imaging (DWI) showed hyperintense lesions, as indicated by the arrows.

**Figure 3:**
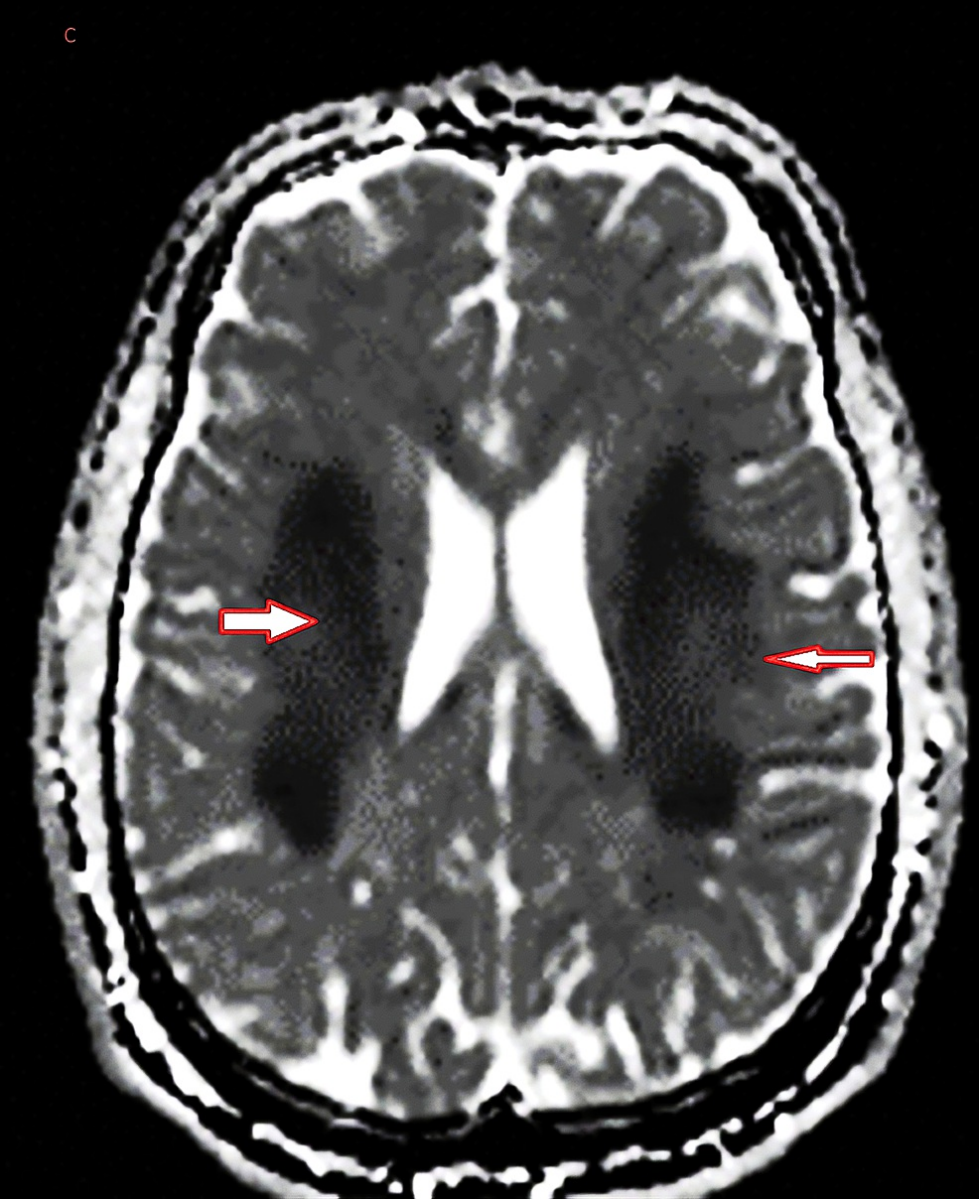
ADC hypointense lesions. The arrows indicate hypointense lesions on the apparent diffusion coefficient (ADC) map.

**Figure 4:**
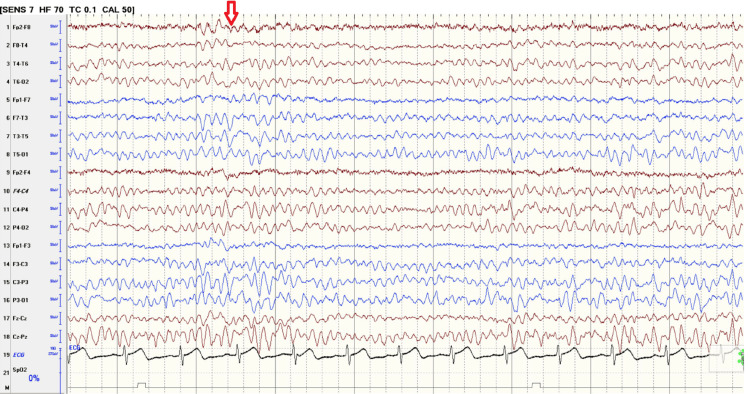
The EEG above shows intermittent slowing of generalized delta-theta activity.

The radiologist reported the plain brain MRI as bilateral symmetrical diffuse T2/FLAIR weighted imaging hyperintensities seen in the white matter of the frontoparietal lobes (Figure 1). Restricted diffusion is denoted by hyperintensity on DWI and hypointensity on the ADC map of the same lesions, suggesting ongoing cytotoxic injury (Figures 2-3). The radiologist's impression was that the MRI findings could suggest acute leukoencephalopathy (Figure 1). EEG showed mild to moderate nonspecific cerebral dysfunction, diffuse and focal over the left posterior quadrant, with no definite epileptiform discharges (Figure 4). Genetic testing confirmed a nonsense mutation c.64C>T (p.Arg22*) in the GJB1 gene. It is worth mentioning that the patient is not following up with his appointments, so some additional investigations were not performed.

## Discussion

This case presentation underscores the atypical presentation of CMTX1, particularly when co-existing with leukodystrophy, highlighting the challenges in diagnosis. CMTX1, caused by mutations in the GJB1 gene, is primarily a peripheral neuropathy, but CNS involvement has been increasingly recognized. Identifying the nonsense mutation c.64C>T (p.Arg22*) in our patient underscores the critical role of genetic testing in confirming the diagnosis and understanding the disease pathology.

The presentation of leukodystrophy in a patient with CMTX1 is rare but significant, expanding the spectrum of neurological manifestations associated with this genetic disorder. Bilateral white matter hyperintensities observed on brain MRI align with reports of CNS involvement in CMTX1, attributed to connexin 32 (Cx32) expression in oligodendrocytes and Schwann cells [2]. This dual expression explains the mixed clinical phenotype of peripheral neuropathy and central white matter changes.

Advances in next-generation sequencing (NGS), particularly whole-exome sequencing (WES), have revolutionized the diagnosis and understanding of genetic neuropathies. These technologies facilitate the identification of novel mutations and provide insights into the genetic burden contributing to phenotypic variability [6]. In our case, the genetic findings highlight the utility of comprehensive genetic analysis in elucidating underlying mutations that influence disease presentation and progression.

Building on the work of Tian D et al. (2020), which explored the demographic and clinical features of 47 CMTX1 patients with episodic CNS deficits, this study aims to investigate the phenotypic spectrum of CMTX1 further. By analyzing a larger cohort or focusing on specific aspects of the disease presentation, we can gain deeper insights into the variability of CMTX1 and its impact on the central nervous system. This knowledge can ultimately improve diagnostic accuracy, guide the development of targeted treatment strategies, and potentially lead to better patient outcomes [5]. These manifestations require careful differential diagnosis to exclude metabolic, autoimmune, and vascular conditions. In our patient, extensive blood work and imaging ruled out these differentials, supporting the diagnosis of CMTX1 with leukodystrophy. Our Neurology team interpreted the episodic attacks seen in the patient as more likely to be seizures due to their episodic nature and total resolution, and previous recent history of generalized tonic-clonic seizure that the patient described. This led to the decision to initiate the patient on oxcarbazepine.

Managing CMTX1 with CNS involvement remains challenging and necessitates a multidisciplinary approach. Neurologists, geneticists, and rehabilitation specialists must work together to address peripheral and central symptoms. Genetic counseling is essential for patient and family education, guiding expectations regarding disease progression and inheritance patterns. Neurorehabilitation is crucial for managing motor and sensory deficits and improving quality of life [3].

Emerging research and clinical trials are exploring targeted therapies to modulate the genetic and molecular pathways involved in CMT, offering hope for more effective treatments [7]. Advances in imaging techniques continue to enhance our understanding of CNS involvement in CMT, guiding more precise diagnosis and management strategies [8].

Lee M et al. found a link between abnormalities detected using diffusion tensor imaging and the severity of the patient's symptoms. This suggests that central nervous system damage might occur alongside peripheral neuropathy in CMT patients [9].

## Conclusions

We report a case of a 23-year-old man with confirmed CMTX1 characterized by white matter lesions caused by a nonsense mutation c.64C>T (p.Arg22*) in the GJB1 gene, highlighting the potential for atypical presentations in CMT. The combination of peripheral neuropathy and central nervous system dysfunction, including seizures and white matter changes on brain MRI, deviates from the classic CMTX1 picture. Further investigations, such as additional genetic testing and brain MRI, are crucial to determine whether these features are related to CMTX1 or represent a co-existing condition. This case underscores the importance of considering a broad differential diagnosis and pursuing a focused approach in managing CMT patients, particularly those with complex presentations.
